# Pilot study to assess visualization and therapy of inflammatory mechanisms after vessel reopening in a mouse stroke model

**DOI:** 10.1038/s41598-017-17533-5

**Published:** 2018-01-15

**Authors:** Ebba Beller, Laura Reuter, Anne Kluge, Christine Preibisch, Ute Lindauer, Alexei Bogdanov, Friederike Lämmer, Claire Delbridge, Kaspar Matiasek, Benedikt J. Schwaiger, Tobias Boeckh-Behrens, Claus Zimmer, Alexandra S. Gersing

**Affiliations:** 1 Department of Diagnostic and Interventional Neuroradiology, Klinikum rechts der Isar der Technischen Universität München, Munich, Germany; 2 Department of Diagnostic and Interventional Radiology, University Hospital Rostock, Rostock, Germany; 3 Translational Neurosurgery, Medical Faculty, RWTH Aachen University, Aachen, Germany; 4 Department of Radiology, University of Massachusetts Medical School, Worcester, MA 01655d USA; 5 Division of Neuropathology, Institute of Pathology, Technical University of Munich, Munich, Germany; 6 Section of Clinical and Comparative Neuropathology, Centre for Clinical Veterinary Medicine, Ludwig-Maximilians-University, Munich, Germany; 7 Department of Radiology, Technical University of Munich, Munich, Germany

## Abstract

After reperfusion therapy in stroke patients secondary inflammatory processes may increase cerebral damage. In this pilot study, effects of anti-inflammatory therapy were assessed in a middle cerebral artery occlusion (MCAO) mouse model after reperfusion. 1 hour after MCAO, the artery was reopened and tacrolimus or NaCl were administered intra-arterially. Perfusion-weighted (PWI) and diffusion-weighted images (DWI) were obtained by MRI during MCAO. DWI, T2- and T1-weighted images with and without Bis-5HT-DTPA administration were obtained 24 hours after MCAO. Neutrophils, Myeloperoxidase-positive-(MPO+)-cells and microglia, including M1 and M2 phenotypes, were assessed immunohistochemically. Treatment with tacrolimus led to significantly smaller apparent diffusion coefficient (ADC) lesion volume within 24 hours (median −55.6mm^3^, range −81.3 to −3.6, vs. median 8.0 mm^3^, range 1.2 to 41.0; P = 0.008) and significantly lower enhancement of Bis-5-HT-DTPA (median signal intensity (SI) ratio_cortex_, median 92.0%, range 82.8% to 97.1%, vs. median 103.1%, range 98.7% to 104.6%; P = 0.008) compared to the NaCl group. Immunohistochemical analysis showed no significant differences between both groups. Intra-arterially administered anti-inflammatory agents after mechanical thrombectomy may improve treatment efficiency in stroke by reducing infarct volume size and MPO activity.

## Introduction

Ischemic stroke is a major health problem and a leading cause of serious long-term disability. Until recently intravenous recombinant tissue plasminogen activator (rt-PA) has been the only proven reperfusion therapy for acute cerebral ischemia^1^. In recent randomized controlled studies, mechanical thrombectomy has become the standard of care in patients with acute ischemic stroke with a proximal large vessel occlusion in the anterior circulation, improving the long-term clinical and functional outcome significantly^2–4^. Yet, successful treatment of acute ischemic stroke still remains a major challenge.

Inflammation and immune responses have been discussed as being important factors in the onset and progression of stroke^5,6^. Within a few hours after onset of ischemia, circulating leukocytes migrate into the brain tissue and release pro-inflammatory mediators, causing secondary damage to tissue within the penumbra^7^. Even reperfusion of ischemic areas, which is critical for saving penumbral tissue, may result in secondary inflammation-mediated ischemia/reperfusion (I/R) injury of the brain tissue^7,8^. Oxidative stress mediators released by inflammatory cells around the I/R injured tissue trigger expression of pro-inflammatory genes^7,8^. As a result, cytokines are upregulated within the cerebral tissue and consequently the expression of adhesion molecules on the endothelial cell surface is induced, mediating the adhesion of leukocytes to endothelia in the ischemic tissue^7,9^. Among the first cells to be recruited are neutrophils^10,11^. One of the major neutrophil effector proteins is the heme-enzyme myeloperoxidase (MPO), which is the most abundant component of azurophilic granules of neutrophils and macrophages^10,12^. During stimulation, myeloperoxidase is secreted by these cells and activates cellular inflammatory signaling cascades^12^. Plasma MPO levels have shown to be elevated in acute stroke patients in comparison to controls^13^. Previous studies have established the MPO-sensitive MR contrast agent (Bis-5-HT-DTPA), enabling the visualization of MPO activity^14–16^.

Cumulative effects of post-ischemic neuroinflammatory changes and I/R injury can lead to dysfunction of the blood-brain barrier, cerebral edema, and neuronal cell death. Therefore, neuroprotective agents that curtail neuroinflammation have become an important area of research in translational medicine^7^. Several studies have shown that tacrolimus, an immunosuppressant widely used to prevent allograft rejection, reduced ischemic injury and ameliorated neurologic deficits in animal models of cerebral ischemia^17–20^. Tacrolimus inhibits the activation of calcineurin, which is activated by excessive influx of Ca^2++^ into cerebral cells during cerebral ischemia, causing the release of inflammatory cytokines and other inflammatory mediators^19^. Tacrolimus is also reported to inhibit perifocal activation of microglial cells. M1 activated microglia produce reactive oxygen species (ROS) and nitric oxide (NO), which damage neuronal cells^21^, whereas M2 activated microglia release anti-inflammatory substances. After stroke induction both phenotypes are expressed but due to the infarction induced tissue damage, microglia is mostly polarized to M1 phenotype^22^. Due to mechanical thrombectomy being the standard of clinical care regarding the therapy of large vessel occlusion, the intra-arterial administration of neuroprotective substances can be easily implemented in clinical routine and therefore should be considered as potential adjunct therapies in the clinical treatment of stroke.

Therefore, in the present study, we assessed neuroprotective effects of intra-arterially administered tacrolimus as kind of an anti-inflammatory model substance in a murine stroke model, utilizing 7 T MR imaging with a MPO-sensitive contrast agent. This agent measures the activity of myeloperoxidase, in order to visualize the anti-inflammatory therapeutic effects after ischemia *in vivo*.

## Results

### MR imaging before and 24 hours after treatment

**Figure 1:**
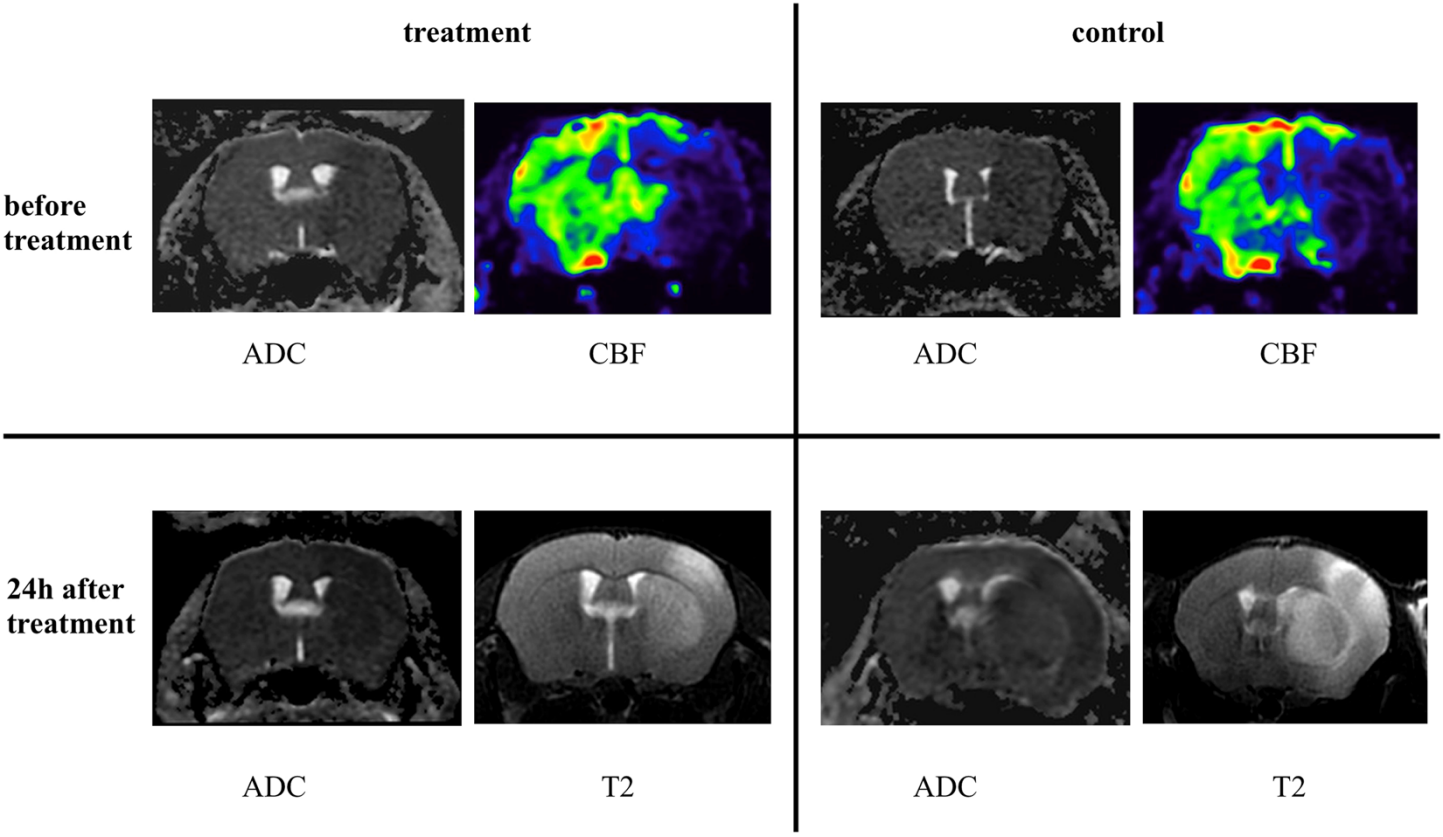
MR imaging before and 24 hours after treatment. Representative baseline ADC and CBF maps (upper row) from a control group (right column) and a treatment group (left column) before treatment, showing no significant differences of the lesion volumes within the right MCA territory. Representative ADC and T2 24 hours after stroke induction (lower row) of a treated mouse (left column) and a control mouse (right column), showing smaller ADC and T2 lesions 24 hours after treatment with tacrolimus compared to the lesions of the NaCl-treated control.

ADC maps and Perfusion maps during 1 hour of MCA occlusion were obtained and only mice with a perfusion deficit were included to receive either 0,6 mg/kg tacrolimus or NaCl only. 24 hours after treatment ADC maps and T2-weighted images were obtained (Fig. 1)

**Figure 2:**
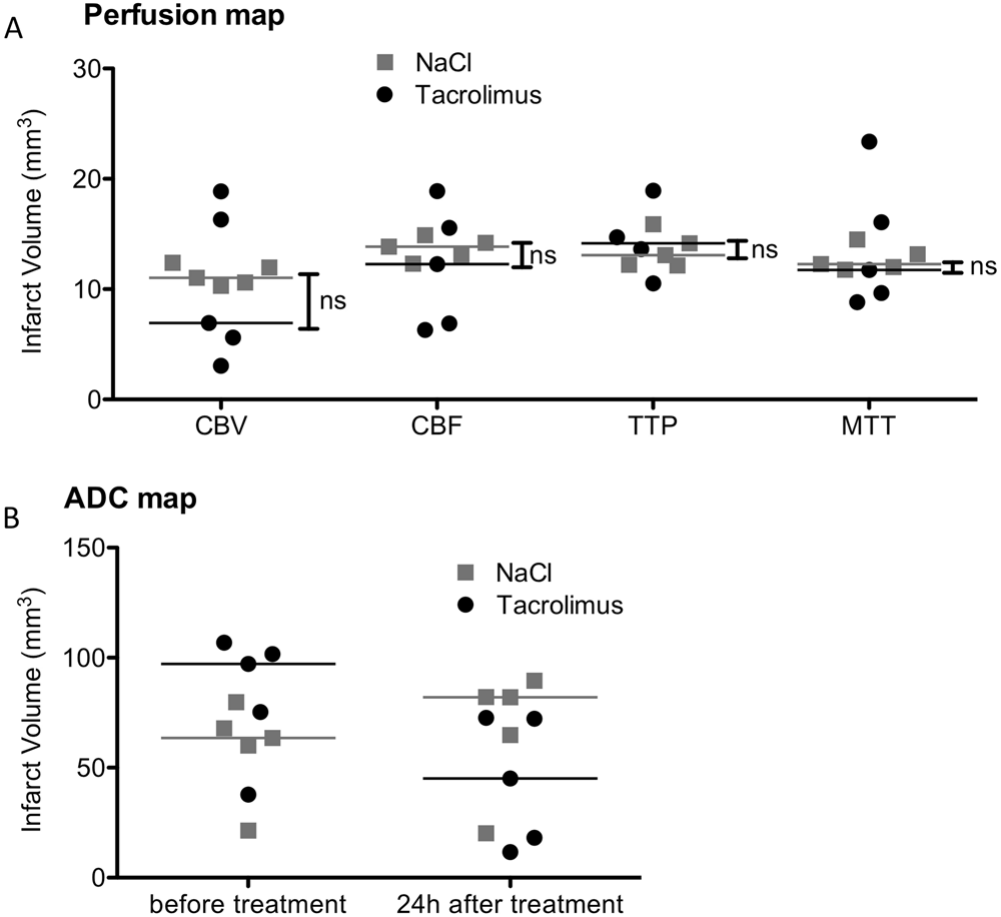
Perfusion and ADC lesion volume in treatment vs. control group. (**A**) MRI perfusion maps CBV, CBF, TTP and MTT did not show a significant difference in decreased perfusion volume between control and treatment group before treatment (CBV: 11.0 [10.3 to 12.4] vs. 6.9 [3.1 to 18.9] mm^3^, P = 0.69, CBF: 13.9 [12.3 to 14.9] vs. 12.3 [6.3 to 18.9] mm^3^, P = 0.69, TTP: 13.0 [12.2 to 15.9] vs. 14.2 [10.5 to 18.9] mm^3^, P = 0,73 and MTT: 12.3 [11.8 to 14.5] vs. 11.7 [8.8 to 23.4] mm^3^, P = 0.69). (**B**) ADC lesion volume had a median volume of 63.5 [21.4 to 79.8] mm^3^ in the control group and 97.2 [37.8 to 106.9] mm^3^ in the treatment group before treatment (P = 0.15). 24 hours after treatment ADC had a lesion volumes of 45.1 mm^3^ [20.2 to 89.5] in the tacrolimus group compared to 82.0 mm^3^ [11.7 to 72.6] in the control group, P = 0.15 (Both graph show median, ns: P = not significant, n = 5 for both groups).

ADC lesion volumes and MR perfusion volume did not show a significant difference between both groups before treatment with tacrolimus or NaCl only. However, ADC lesion volume and decreased perfusion volume showed a large variation within the groups. Both T2 and ADC showed smaller lesion volumes 24 hours after MCAO in the tacrolimus group compared to the control group (44.4 mm^3^ [8.7 to 99.0] vs. 81.8 mm^3^ [12.4 to 94.5], P = 0.84 and 45.1 mm^3^ [20.2 to 89.5] vs. 82.0 mm^3^ [11.7 to 72.6], P = 0.15), yet the comparison of these results between the groups did not reach the level of significance (Fig. 2). There was also no significant difference in the functional neurological examination with the 14-point neuroscore mNSS 24 hours after stroke induction between the treatment group (9 point [6 to 14 points] and the control group (10 points [6 to 12 points]), (P = 0.92).

**Figure 3:**
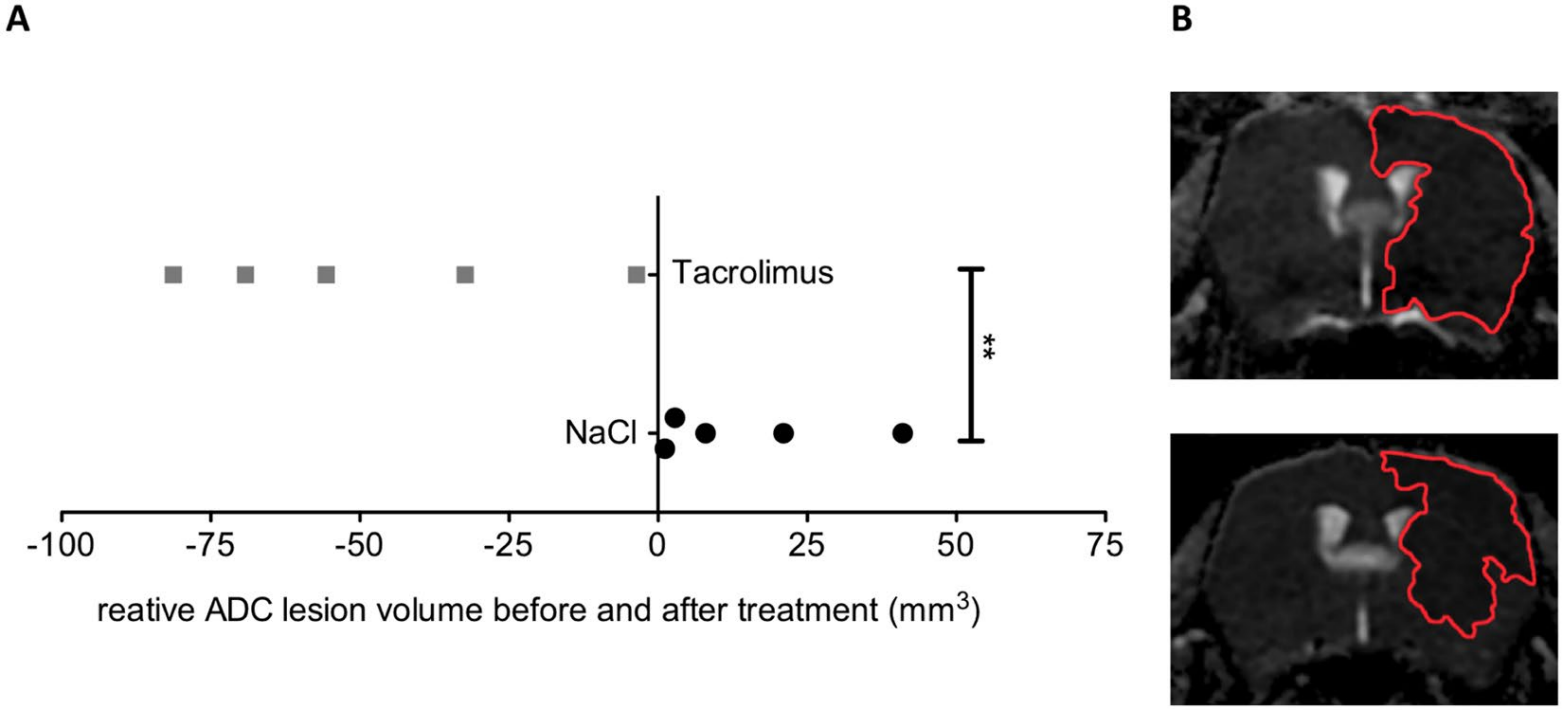
Comparison of change of ADC lesion volume before and 24 hours after treatment between the tacrolimus group and the control group. (**A**) Change of ADC lesion volume at baseline and 24 hours after treatment with tacrolimus showed a ADC lesion volume reduction (−55.6 mm^3^ [−81.3 to −3.6], whereas the control group showed an increase of the ADC lesion volume (8.0 mm^3^ [1.2 to 41.0]), resulting in a significant difference between both groups (**P = 0.008) (n = 5, both groups) (**A**). Representative ADC lesion volume at baseline (upper row) and 24 hours after treatment with tacrolimus (lower row), showing a significantly smaller ADC lesion volume after treatment with tacrolimus compared to the baseline ADC lesion volume (**B**).

The analysis of the change of the ADC lesion volume before and 24 hours after tacrolimus treatment compared to the change of the ADC lesion volume of the control group between the same time points was significantly different (**P = 0.008), showing a reduction of the mean ADC lesion volume in the tacrolimus treatment group (−55.6 mm^3^ [−81.3 to −3.6]), compared to an ADC lesion volume increase in the control group (8.0 mm^3^ [1.2 to 41.0]), (Fig. 3).

### Significant lower Signal Intensity of Bis-5HT-DTPA in the Treatment Group

**Figure 4:**
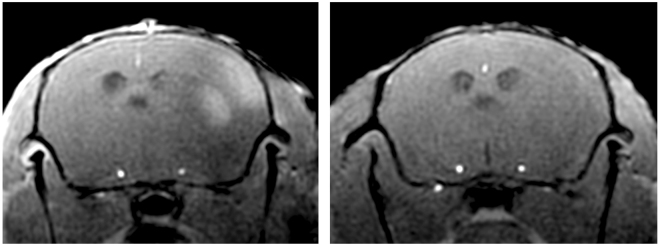
1 hour after Bis-5HT-DTPA administration, MRI signal enhancement was observed in the ischemic right MCA territory. Representative MRI images of T1 after Bis-5HT-DTPA i.v. from a mouse of a control group (left) and the treatment group (right) with contrast enhancement in the right MCA territory 24 hours after stroke induction.

**Figure 5:**
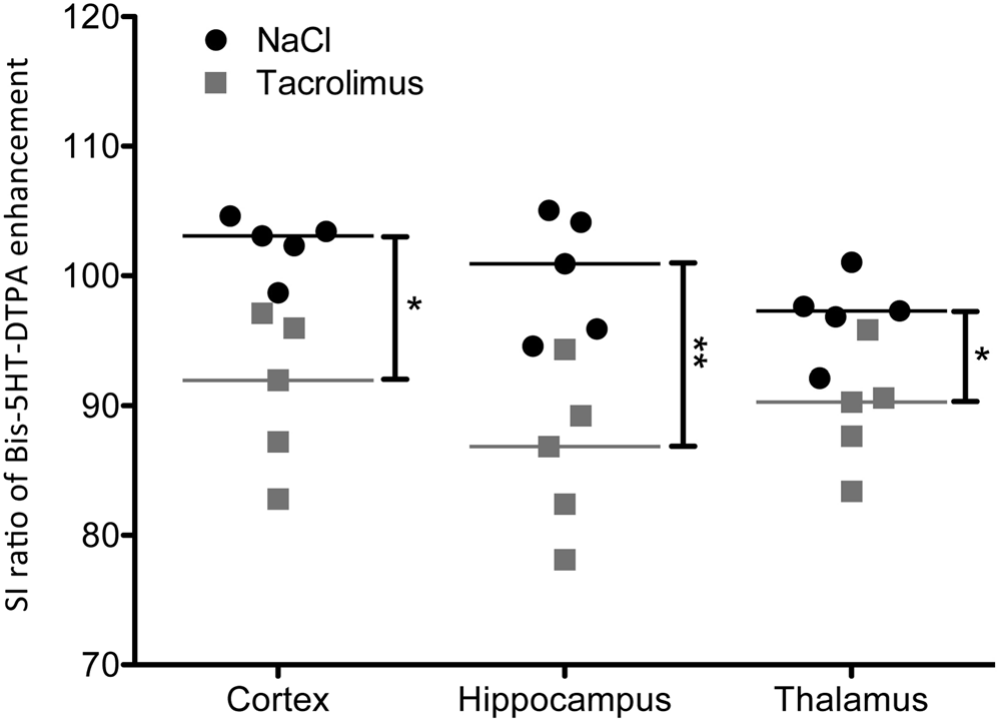
Significantly lower SI ratio of Bis-5HT-DTPA enhancement in cortex, hippocampus and thalamus in the group treated with tacrolimus compared to the control group without anti-inflammatory treatment. Quantitative analysis of Bis-5HT-DTPA enhancement by comparing signal intensity (SI) ratio between the ischemic region and the contralateral ROI mirrored along the midline of the group treated with tacrolimus and the control group (n = 5, each group) in cortex, hippocampus and thalamus (graph shows median, **P = 0.008 and *P = 0.016).

Quantitative analysis of Bis-5HT-DTPA enhancement in the ischemic area was conducted by measuring mean signal intensity in cortex, hippocampus and thalamus and dividing the results of the ischemic hemisphere by that of the non-ischemic hemisphere (Fig. 4). The median SI ratio in the cortex was 92.0 [82.8 to 97.1] in the treatment group compared to 103.1 [98.7 to 104.6] in the control group (**P = 0.008). Comparable differences were found in the hippocampus (tacrolimus treatment group: 86.9 [78.1 to 94.3]; control group: 100.9 [94.6 to 105.1], (**P = 0.008)) and in the thalamus (tacrolimus treatment group: 90.3 [83.4 to 95.8]; control group: 97.3 [92.1 to 101.0], (*P = 0.016; Fig. 5)), suggesting lower MPO-activity within the tacrolimus treatment group compared to the control group without anti-inflammatory treatment.

### Immunohistochemical analysis of anti-inflammatory effects of tacrolimus after stroke

**Figure 6:**
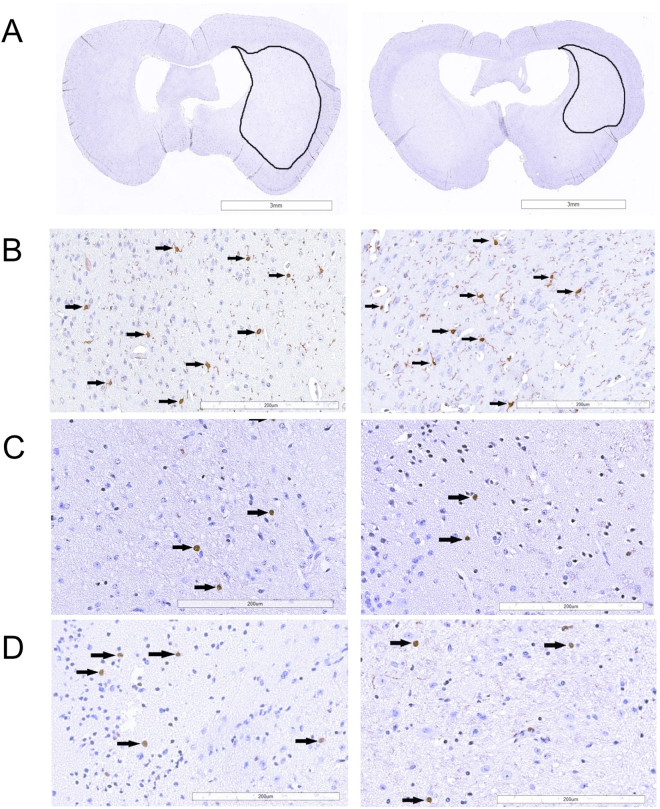
Microglial and neutrophil response and recruitment of MPO-positive cells after stroke induction. Representative formalin-fixed, paraffin-embedded mouse brain tissue sections (2 µm) after immunohistochemical (IHC) staining 24 hours after stroke induction in a non-treated mouse (left column) and after treatment with tacrolimus (right column). Sections are shown in 1x magnification with encircled infarct area (**A**). Microglia response was analyzed by quantifying Iba1 (**B**). Neutrophils and MPO-positive cells were detected by antibodies against Ly-6G/Ly-6C and MPO (C + D). Positive cells are marked with arrows.

Measurements of the infarct size in the HE-stained sections corrected for edema showed no significant difference between treatment group (15.1% [6.4 to 38.6%]) and control group (23.4% [6.3 to 28.4%]), (P = 1.0). In addition to infarct size, parenchymal infiltration of microglia, extravascular neutrophils and MPO-positive cells were immunohistochemically analyzed (Fig. 6A–C).

**Figure 7:**
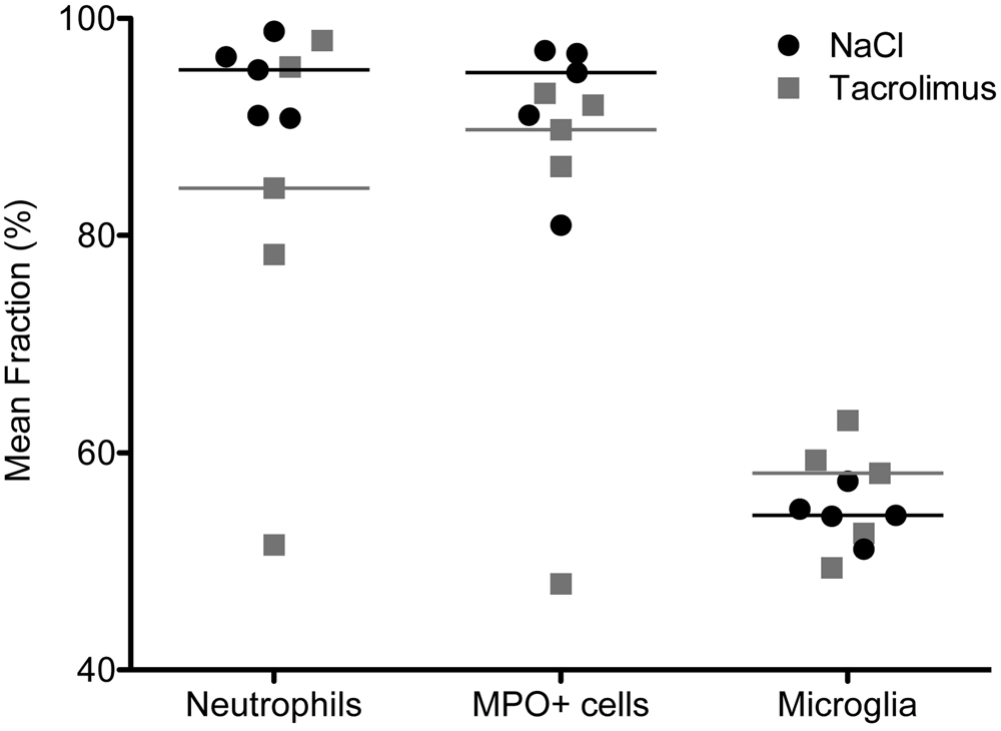
Neutrophil infiltration, microglial response and recruitment of MPO-positive cells after stroke induction in the treatment group compared to the control group. Quantification of neutrophils, MPO-positive cells and number of pixel count in the infarcted area is presented in percent of the total number of cells/pixels in both hemispheres (graph shows median), (n = 5, both groups). Neutrophils and MPO+ cells showed less infiltration of the infarcted area in the treatment group compared to the control group (P = 0.30 or P = 0.22, respectively). Microglia response showed no significant difference between both groups, P = 0.5.

Quantitative analysis of neutrophils and MPO + cells showed a reduction in infiltration of these cells in the ischemic area in the treatment group compared to the control group (84.4% [51.5 to 98.0%] vs. 95.3% [90.8 to 98.8%], P = 0.30 and 89.7% [48.0 to 93.1%] vs. 95.0% [81.0 to 97.0%], P = 0.22, respectively; Fig. 7). There was no significant difference of the microglial response between the group treated with tacrolimus (58.1% [49.4 to 63.0%] and the control group (54.2% [51.1 to 57.4%], P = 0.5; Fig. 7).

**Figure 8:**
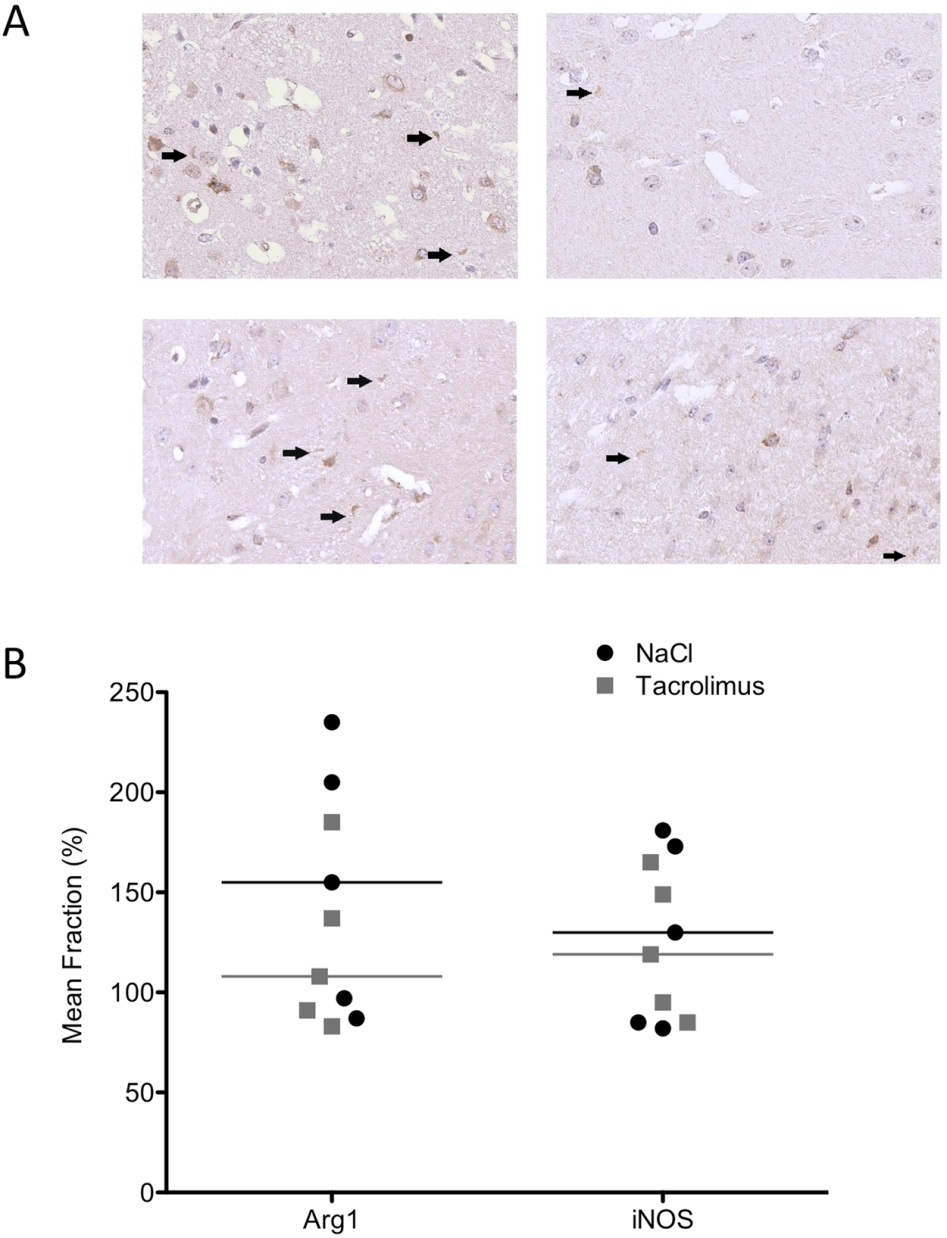
Microglial phenotypes. Representative images of iNOS staining images (left column) and Arg1 staining images (right column) of a mouse treated with tacrolimus (upper row) and of the control group (lower row). Images are shown in 20x magnification and positive stained cells are marked with arrows. Quantitative analysis showed no significant difference of the M1 and M2 microglial response between the group treated with tacrolimus and the control group (M1/iNOS: 119.3% [85.2 to 165.5%] and 130.2% [81.7 to 181.1%], P = 1.0; M2/Arg1: 108.3% [82.6 to 185.3%] and 154.5% [87.0 to 235.0%], P = 0.4), (**B**).

Additional histological analyses were performed using iNOS and Arg1 to differentiate between M1 and M2 activated microglia (Fig. 8A). Additional histological analyses were performed using iNOS and Arg1 to differentiate between M1 and M2 activated microglia (Fig. 8A). There was no significant difference of the M1 (P = 1.0) and M2 (P = 0.4) microglial response between the group treated with tacrolimus and the control group (Fig. 8B).

## Discussion

Preliminary results showed that intra-arterial treatment with tacrolimus leads to a significant reduction of the ADC lesion volume within 24 hours as well as to significantly lower enhancement of Bis-5-HT-DTPA compared to the control group, suggesting less activity of myeloperoxidase and infarct volume reduction after tacrolimus treatment. Immunohistochemical analysis showed less infiltration of neutrophils and MPO+ cells in the ischemic area after treatment with tacrolimus compared to the controls.

Different mechanisms are involved in the pathogenesis of stroke, but increasing evidence suggests that one of the processes worsening clinical outcome is early inflammation with the synthesis and the release of pro-inflammatory cytokines that activate several cells contributing to the progression of brain injury^23^. In particular, neutrophil granulocytes and microglia have been considered to be the main players in post-ischemic infarction^24^. Targeting specific inflammatory or immune pathways therefore represents a treatment strategy in acute ischemic stroke especially after successful reperfusion (e.g. through thrombectomy), which may be of importance in the future.

Despite the favorable effect of neuroprotective agents in animal stroke models, such anti-inflammatory substances have not yet been approved due to the lack of evidence of sufficient efficacy and good safety in clinical studies^25^. Hence, intra-arterial application of neuroprotective agents, e.g. tacrolimus, could be implemented easily in the clinical routine in combination with mechanical thrombectomy. Several animal studies have successfully shown that intravenous therapy with tacrolimus reduced ischemic injury and ameliorated neurologic deficits in animal models of cerebral ischemia^17–20^. Intra-arterial injections of immunosuppressive agents like tacrolimus in a reopened vessel may improve the treatment efficiency in a clinical setting by increasing the dose for the infarcted volume, while simultaneously causing the least detrimental effect on healthy tissue and organs. Instead of tacrolimus i.v. injections with a dosage of 1 mg/kg as reported in previous studies^17,19,20^, it would be possible to maintain the therapeutic range of tacrolimus with dose reduction of 30% in patients in which the substance is delivered intra-arterially and therefore locally^26^. Therefore intra-arterial application of neuroprotective agents after mechanical thrombectomy may also have the potential to become a promising strategy for avoiding adverse effects.

Among the first cells to infiltrate areas of brain ischemia are neutrophils^7^, with myeloperoxidase being one of the major neutrophil effector proteins and being the most abundant component of azurophilic granules^10,12^. Enzymatic activity of neutrophilic myeloperoxidase can therefore be used as a highly selective and sensitive target for detecting neuroinflammation by MR imaging *in vivo*^27^. Breckwoldt *et al*. could show that MPO is widely distributed in ischemic tissues and in contrast to the conventional MRI contrast agent Gd-DTPA, which images blood–brain barrier disturbance, Bis-5HT-DTPA is able to additionally visualize MPO activity and confirm inflammation *in vivo*^14^. In our study the analysis of Bis-5HT-DTPA enhancement showed that the signal intensity ratio was significantly lower in the group treated with tacrolimus compared to the control group and therefore reflected the ability of anti-inflammatory treatment with tacrolimus to reduce the inflammation process in the ischemic area. Therefore MPO imaging could potentially be used in a clinical setting to validate therapeutic effects of anti-inflammatory treatment in stroke patients.

One limitation of our pilot study is the small number of animals in the treatment and control group due to the high mortality rate for the mice in this experimental setting. Therefore further studies with a larger sample size are required to confirm our results. Yet, even this small number of animals showed significant differences of the change of the ADC lesion volumes before and after tacrolimus treatment compared to NaCl treated animals. In this study we did not measure blood pressure and blood gas during stroke induction. This limitation could affect post-stroke development and size due to inter-individual vulnerability of the animals to periprocedural complications such as hypotension or hypoxemia^28^. However, a major strength of this study is that DW imaging was obtained not only 24 hours after but also during the 1 hour of MCA occlusion. DW imaging can detect brain ischemia induced diffusion restriction in the early phase in its full extent, thus ensuring an estimation of the lesion size induced by ischemia^29^. Therefore DWI was performed to acquire the *in vivo* lesion size before therapeutic regimen was started and lesion sizes were matched and distributed to the treatment and control group. By that, heterogeneity of lesion size volumes between the groups was reduced^29^. Even with this relatively small number of animals, the relative changes of the ADC volume before and after treatment/NaCl were significantly lower in the treatment group compared to the control group.

Another important limitation is the short follow-up time of 24 hours since the infarct size is typically not fully developed until 72 hours post-stroke in rodents. Besides our results showed less infiltration of neutrophils and MPO+ cells in the infarcted area in the treatment group compared to the controls whereas there was no significant difference found regarding the microglia. Recent studies indicate that the number of neutrophil granulocytes peaks between day 1 and 3 after experimental stroke, while microglia was found to peak later than day 3 and stay in the infarcted area for longer time periods^24,30^. Although neutrophils and microglia show a steady increase in cellular infiltration from day 1 on^24^, the short treatment duration of 24 hours might have been the reason why we did not detect a significant treatment effect histologically. There was also no significant difference in the functional neurological examination between treatment and control group. This could be due to the initial brain tissue swelling during the acute phase of ischemic stroke in both groups^31,32^. However, whether there are differences in microglia infiltration and functional outcome between both groups after longer periods of follow-up time after treatment have to be confirmed by further studies.

In conclusion, in this pilot study intra-arterial treatment with tacrolimus lead to a significant reduction in infarct volume over 24 hours, which might be caused by the anti-inflammatory effect on inflammation-mediated ischemia/reperfusion injury. These anti-inflammatory effects can be visualized by utilizing Bis-5-HT-DTPA, which measures the activity of myeloperoxidase, showing significantly less enhancement in the group treated with tacrolimus compared to the control group without anti-inflammatory treatment. Immunohistochemical analysis showed decreased infiltration of neutrophils and MPO+ cells in the infarcted area after treatment. Therefore our study could be used as a model for prospective local anti-inflammatory therapeutic approaches in stroke by applying the technique of intra-arterial injections after mechanical thrombectomy with restored tissue perfusion.

## Method

### Experimental Stroke Model and Intra-arterial Treatment with Tacrolimus

All experiments were conducted in accordance with government and institutional animal welfare guidelines (FELASA). The study was approved by the local government (Regierung von Oberbayern Gz. 55.2-1-54-2532-159-13). C57/BL6 male mice (26 g +/− 1,2 g) were ordered from Charles River Deutschland GmbH at the age of 11 to 12 weeks, housing in groups of four or five. Environmental conditions were a temperature of 22 °C ± 2°, humidity of 55% ± 10%, lighting of 40–140 lux and a 12:12 light:dark cycle with lights on at 0600 and off at 1800. Animals were housed in 369 × 165 × 132 (L × W × H) mm cages (Tecniplast UK, 1145 T, Polycarbonate) and given access to mouse maintenance food (#1324 Altromin, Spezialfutter GmbH&Co. KG, Lage, Germany) and water ad libitum.

Mice were anesthetized with a combination of medetomidine (0,5 mg/kg), midazolam (5 mg/kg) and fentanyl (0,05 mg/kg) (MMF). Middle cerebral artery occlusion (MCAO) was induced with a silicone-coated 6.0 nylon monofilament for 1 hour as described previously^33,34^. After 1 h of occlusion the nylon thread was removed to initiate reperfusion. Mice were randomly divided into two groups. A catheter with an outer diameter of 0,267 mm was inserted into the common carotid artery (CCA) through a second puncture, and the catheter tip was placed at the level of the carotid bifurcation. In the treatment group 0,6 mg/kg tacrolimus in 0,03 ml NaCl was injected intra-arterially over the course of 30 seconds. The control group received an equivalent volume of NaCl only. Core temperature was maintained at 36.5 ± 0.5 °C throughout the experiment. Heart rate (HR) and breathing rate (BR) were also measured during the experiment and were kept as constant as possible by adjusting anesthesia.

Stroke induction was performed in 28 mice. Death due to intraoperative complications occurred in six (21%) mice with middle cerebral artery occlusion. Two (7%) mice of both treatment and control group died after/while receiving NaCl/tacrolimus i.a. Six (21%) mice were excluded due to lack of lesion on perfusion MR imaging. Two (7%) mice were excluded due to failed puncture of the tail vein, which is why application of contrast enhancement was not possible. Therefore complete MR and histology datasets were acquired from 10 mice in total (tacrolimus group: n = 5; NaCl control group: n = 5).

### Magnetic Resonance Imaging

Utilizing a 7 T small-animal MRI scanner (GE/Agilent MR901, GE Healthcare/Agilent Technologies) MRI examinations were conducted during 1 hour of MCA occlusion and 24 hours after vessel occlusion. The effectiveness of the anesthesia was controlled initially by testing the corneal reflex, reaction of the hind legs against pressure and during the scan by measuring the heart rate (HR) and breathing rate (BR). Mice were placed in prone position in a U-shaped rail equipped with integrated heating. HR and BR were monitored throughout the MR examination. For administration of contrast agent, a polyurethane catheter capped with a needle port was placed in the tail vein.

The MRI protocol obtained during the one hour of MCA occlusion, included the following sequences (FOV: 2 cm × 2 cm; 0,5 cm thickness): a diffusion-weighted imaging (DWI) sequence (Matrix: 128 × 64 (Phase FOV: 50%), TE: 33.7 ms; TR; 3000 ms; FA: 90; b-values of 0 and 800 s/mm^2^); a perfusion sequence (T2*weighted Dynamic Susceptibility Imaging; Matrix: 128 × 96 (Phase FOV: 75%), TE: 15.2 ms; TR: 552 ms; FA: 30; 2-shot EPI, 192 temporal positions; FOV: 2 cm × 2 cm; 0,5 cm thickness). Only mice with a lesion on perfusion MR imaging were included into the study.

The MRI protocol obtained 24 hours after MCAO included the following sequences (FOV: 2 cm × 2 cm; 0,5 cm thickness): a DWI (see above); a T2-weighted sequence (Matrix: 192 × 192, TE: 45 ms; TR: 3500 ms; FA: 90; NEX [Number of Excitations]: 2; ETL [Echo Train Length]: 4; FOV: 2 cm × 2 cm; 0,5 cm thickness); a T1-weighted sequence (Matrix: 160 × 160, TE = 2 ms; TR = 15 ms; FA = 15; NEX = 3; FOV: 2 cm × 2 cm; 0,5 cm thickness) was acquired before and 1 hour after the administration of MPO-sensitive contrast agent (Bis-5HT-DTPA). Bis-5-HT-DTPA measures the activity of myeloperoxidase *in vivo*, which can serve as a surrogate for the intensity of leukocyte influx^12,35,36^ and was administered i.v. at a dosage of 0.3 mmol Gd/kg bodyweight 24 hours after MCAO. Stocks were prepared as 8 mg of the contrast agent and dissolved in 0,15 ml sterile injection buffer (5% dimeglumine in Dulbecco’s phosphate buffered saline (DPBS), pH 7.5).

### Image analyses

Parameter maps as apparent diffusion coefficient (ADC) and perfusion maps were generated in MATLAB R2013a (MathWorks, Natick, US) using standard analyses algorithms^37^. A noise-level threshold of b = 0 was utilized to mask the ADC images and Wiener filtering was applied for denoising. All images were analyzed using OsiriX (OsiriX Foundation, Geneva, Switzerland). Lesions on DW- and perfusion-weighted images were delineated manually on every axial slice using the OsiriX closed polygon tool. The lesions were defined as tissue with decrease in perfusion or ADC decrease of two or more SDs outside the mean of the contralateral hemisphere, as described previously^38^. Quantitative analysis of Bis-5HT-DTPA enhancement was conducted by using operator-defined region-of-interest (ROI) measurements of mean signal intensity. ROIs were placed around cortex, hippocampus and thalamus. The signal intensity ratio between ischemic injury and normal brain was calculated by dividing the mean signal intensity of cortex, hippocampus and thalamus on the ischemic hemisphere by that of the non-ischemic hemisphere. Two blinded readers (E.B. and L.R.) evaluated lesion volume on DWI/PWI imaging and T2-weighted imaging and quantitative analysis of Bis-5HT-DTPA enhancement in consensus reading.

### Evaluation of neurological function

Neurological function was evaluated directly prior to the stroke surgery and at 24 h post-MCAO before the second MRI on a 14 point scale neuroscore mNSS modified by Cai *et al*.^39^, testing hemiparesis, gait, coordination and sensory functions.

### Immunohistochemical staining and quantification

Mice were sacrificed 24 hours after stroke using an overdose of the combination of MMF and Narcoren i.p. After intra-cardiac perfusion with phosphate-buffered saline (PBS), the brains were removed, placed into paraformaldehyde (10%) over night and were then embedded into paraffin for histological and immunohistochemical investigation. Paraffin sections were cut in coronal plane with a thickness of 2 μm. Standard sections were stained with hematoxylin-eosin. For immunohistochemical staining the sections were deparaffinized for 30 minutes, using xylene and a series of graded alcohols until they were fully hydrated in ultrapure water. Next, they were boiled in citrate buffer at pH 6.0 for 30 minutes. Endogenous peroxidase was blocked with a 1.5% hydrogen peroxide for 20 minutes and endogenous biotin was blocked right after in blocking buffer (1% BSA, 0.2% fish gelatin, 0,02% sodium acid and 0.1% Triton X-100 in PBS) with avidin (2,5%). Antibodies against Ly-6G/Ly-6C (Neutrophil Marker 6A608: sc-71674, Santa Cruz Biotechnology), Iba1 (Anti-Iba 1, Wako Chemicals, Japan), and MPO (#RB-373-A, Thermo Scientific™) were diluted (1:250), (1:500) and (1:100) in blocking buffer with biotin (2,5%) overnight, followed by biotinylated anti-rat secondary antibody made in goat (neutrophils) or biotinylated anti-rabbit secondary antibody made in horse (MPO and Iba1) in blocking buffer (1:400) for 30 minutes. Staining was visualized with the ABC-DAB system (Vector Laboratories, Burlingham, CA, USA), followed by a 2 min incubation of hematoxylin as the counterstain. After staining, the slides were dehydrated in graded alcohols, cleared in xylene and coverslipped.

For the evaluation of microglia phenotypes, we used Immpress instead of ABC system for the immunohistochemical analysis. Sections were incubated at 37 °C overnight, afterwards deparaffinized for 65 minutes, using xylene and a series of graded alcohols until they were fully rehydrated in ultrapure water. Sections were boiled in citrate buffer (iNOS) or Tris-EDTA (Arg1) for 20 minutes. Endogenous peroxidase was blocked with hydrogen peroxide for 30 minutes and after that endogenous biotin was blocked with Immpress Reagent Horse (R. T. U. normal horse serum 2,5%, Vector Laboratories, Burlinghame, CA, USA). Antibodies against Arginase 1 (anti-liver Arg1, ab133543, Abcam, Cambridge, UK) and inducible Nitricoxidsynthase (anti-iNOS, ab136918, Abcam, Cambridge, UK) were diluted (1:250 or 1:500, respectively) in PBS and incubated overnight. Immpress Reagent Rabbit (Immpress reagent ant-rabbit Ig, Vector Laboratories, Burlinghame, CA, USA) as secondary antibody was applied for 30 minutes, followed by DAB system (Vector Laboratories, Burlinghame, CA, USA) to visualize staining. After a short incubation in hematoxylin as the counterstain, sections were dehydrated in graded alcohols and xylene and coverslipped. To differentiate between microglia and astrocytes, one section out of ten was used for double labeling with iNOS and S100.

Sections were scanned using Aperio AT2 Console (Leica Biosystems Imaging Inc., Nussloch, Germany). The acquired digital images were analyzed by applying the Image Scope analysis software to quantify Iba1 with automated image analysis algorithm Aperio Positive Pixel Count (version 12.2.2.5015; Aperio Technologies, Vista, CA, United States). Extravascular neutrophils and MPO-positive cells were quantified by manually counting the immunoreactive cells in the ischemic hemisphere and the non-ischemic hemisphere in the 10 successive sections corresponding to the largest cross-sectional lesion size on diffusion-weighted MRI 24 hours after MCAO. Quantification of neutrophils, MPO-positive cells and number of pixel count in the infarcted area is presented in percent of the total number of cells/pixels in both hemispheres. In the HE-stained sections the cross-sectional infarct lesion was delineated and infarct volume was calculated by multiplying the cross-sectional infarct area by section thickness. Edema corrected infarct size was calculated (corrected infarct volume = measured infarct volume × [volume of non-ischemic hemisphere/volume of ischemic hemisphere]) and is presented in percent of the total volume of the contralateral hemisphere to correct for inter-individual differences in brain size.

### Statistical Analysis

Results are reported as median and range and the manuscript was written in accordance with ARRIVE guidelines^40^. Statistical comparisons were performed using two-tailed non-parametric Mann-Whitney-U test for independent samples. Statistical analysis was performed using GraphPad Prism software 167 (version 5.0a). A p-value < 0.05 was considered to indicate statistical significance.
